# Protective effects of zingerone against sodium arsenite-induced lung toxicity: A multi-biomarker approach

**DOI:** 10.22038/IJBMS.2023.71905.15623

**Published:** 2023

**Authors:** Hasan Şimşek, Sefa Küçükler, Cihan Gür, Mustafa İleritürk, Serpil Aygörmez, Fatih Mehmet Kandemir

**Affiliations:** 1 Department of Physiology, Faculty of Medicine, Aksaray University, Aksaray, Türkiye; 2 Department of Veterinary Biochemistry, Faculty of Veterinary, Atatürk University, Erzurum, Türkiye; 3 Department of Animal Science, Horasan Vocational College, Ataturk University, Erzurum, Türkiye; 4 Department of Veterinary Biochemistry, Faculty of Veterinary, Kafkas University, Kars, Türkiye; 5 Department of Medical Biochemistry, Faculty of Medicine, Aksaray University, Aksaray, Türkiye

**Keywords:** Apoptosis, Autophagy, Inflammation, Lung, Oxidative stress, Sodium arsenite, Toxicity, Zingerone

## Abstract

**Objective(s):**

Sodium arsenite (SA) exposure is toxic to the body. Zingerone (ZNG) is a flavonoid with many biological properties found naturally in honey and plants. This study aimed to determine the effects of ZNG on SA-induced rat lung toxicity.

**Materials and Methods:**

Thirty-five male Sprague rats were divided into Control, SA, ZNG, SA+ZNG25, and SA+ZNG50 groups (n=7). SA 10 mg/kg and ZNG were administered at two doses (25 and 50 mg/kg) (orally, 14 days). Analysis of oxidative stress, inflammation damage, apoptosis damage, and autophagic damage markers in lung tissue were determined by biochemical and histological methods.

**Results:**

The administration of ZNG reduced oxidative stress by increasing SA-induced decreased antioxidant enzyme activities, increasing Nrf-2, HO-1, and NQO1, and decreasing MDA level. ZNG administration reduced inflammation marker levels. Anti-apoptotic Bcl-2 increased and apoptotic Bax and Caspase-3 decreased with ZNG. ZNG promoted the regression of autophagy by reducing Beclin-1, LC3A, and LC3B levels.

**Conclusion:**

Evaluating all data showed that SA caused toxic damage to lung tissue by increasing inflammation, apoptosis, autophagy, and oxidant levels, whereas ZNG had a protective effect by reducing this damage.

## Introduction

Heavy metals have become a major concern for human health and environmental toxicology as a result of advances in agriculture and industry, owing to their increasing bioaccumulation, toxicity, and persistence in aquatic ecosystems (1). Arsenic (AR), which ranks 20th in terms of its presence in soil, is one of the toxic heavy metals with the highest environmental or occupational exposure (2, 3). Long-term exposure to AR, particularlye from drinking water, is a public health hazard. Studies have reported that around 200 million people are chronically exposed to toxic levels of AR (4). Even at low concentrations, AR causes toxicity (5). AR exposure is known to induce reactive oxygen species (ROS) generation resulting in lipid peroxidation (LP), protein oxidation, and DNA damage, the mechanisms underlying AR toxicity have not yet been fully elucidated (6).

Moreover, AR compounds are also dangerous and are among the toxic substances known to be carcinogenic to humans. Sodium arsenite (SA) is the most dangerous among these compounds to human and animal health (7). AR compounds are used in the production of specialty glass, wood preservatives, herbicides, insecticides, and drugs used to treat blood cancer (8). Arsenite, a toxic inorganic trivalent arsenic compound (As3+) with potent toxicity, has a direct affinity for the sulfhydryl group and causes a significant increase of ROS in many tissues (9). Exposure to toxic substances such as As3+ induces oxidative stress (OS) damage due to increased ROS and therefore anti-oxidants may be required to maintain oxidant/anti-oxidant homeostasis (5). Recent research has determined that natural plant-derived substances are effective in reducing AR toxicity in various organs and tissues (6).

Flavonoids are plant anti-oxidants that show biological and pharmacological properties in many cell types *in vivo *and* in vitro* (10). One of these flavonoids, zingerone (ZNG), is released in the dried form of ginger and during the contact of gingerols with heat, and its most remarkable feature is that it is a powerful anti-oxidant (11). Aside from its anti-oxidant properties, ZNG also affects signaling pathways involved in many cellular metabolic pathways. Previous research has reported that ZNG has many important biological activities, especially anti-oxidant properties (12). ZNG presents these effects by reducing ROS production, suppressing inflammatory cytokine transcription, inhibiting Nuclear factor kappa-B (NF-κB), inhibiting apoptotic pathways, and up-regulating anti-oxidants (13).

Studies to minimize heavy metal-induced toxicity damage, which has increased due to increased environmental pollution, have recently come to the fore. In this context, this study aimed to determine the curative effects of ZNG, a plant-derived substance, on SA-induced lung toxicity. For this purpose, the effects of ZNG on OS damage caused by SA in lung tissue and inflammation, apoptotic, autophagy, and LP damages triggered by this damage were determined by biochemical, molecular, and histological methods.

## Materials and Methods

***Chemicals***

SA, ZNG, and all other chemicals were obtained from Sigma (MO, USA). All chemicals were of analytical purity. 

***Animals and experimental protocol***

Thirty-five Sprague Dawley rats (220–250 g, male, 12-14 weeks) were employed in the experiments. Rats were obtained from Atatürk University Laboratory Animal Centre (Erzurum, Turkey). Rats were housed under ventilated standard conditions of 12 hr of light/dark, 45 ± 5% humidity, and 23±2 °C. Rats had free access to feed and water. They were randomly divided into five groups (n=7). SA and ZNG doses were determined from the literature (5, 12, 14).

Control: Saline was given orally once a day for 14 days.

Sodium Arsenite (SA): SA was given orally at a dose of 10 mg/kg for 14 days.

Zingerone (ZNG 50 mg/kg): 50 mg/kg dose was given orally for 14 days.

Sodium Arsenite +Zingerone 25 mg/kg (SA+ZNG25 mg/kg): SA was given at a dose of 10 mg/kg followed by ZNG 25 mg/kg orally for 14 days.

Sodium Arsenite +Zingerone 50 mg/kg (SA+ZNG50 mg/kg): SA was given at a dose of 10 mg/kg followed by ZNG 50 mg/kg orally for 14 days.

***Collection of samples***

Rats were decapitated under anesthesia (mild sevoflurane) 24 hr after the last ZNG administration (day 15). Lung tissues were then collected. They tissues were separated from the surrounding tissues, washed with cold saline, and divided into two parts. One part was stored at -80 °C for biochemical and molecular analysis. The other part was placed in a 10% formaldehyde solution for histopathologic examination.

***Analysis of lipid peroxidation***

LP levels of lung tissues were determined with malondialdehyde (MDA) level. To determine the level of MDA, it was reacted with thiobarbituric acid, and the color formed was measured by absorbance at the 532-nanometer wavelength. Lung tissues were homogenized with 1.15% potassium chloride using a Tissue Lyser II homogenizer (Qiagen, The Netherlands). The homogenates obtained were centrifuged at 4 °C and 1,000xg for 15 min, and the supernatant was collected and used in the analysis. MDA levels were determined according to the previous study (15).

***Anti-oxidant analysis***

Catalase (CAT), superoxide dismutase (SOD), glutathione peroxidase (GPx) activities, and glutathione (GSH) levels were measured for the anti-oxidant status of lung tissues. The supernatants used for the analysis of CAT, SOD, GPx, and GSH were obtained in a similar manner as in LP. CAT, SOD, GPx, and GSH were determined according to the methods of Aebi (16), Sun *et al*. (17), Lawrence and Burk (18), and Sedlak and Lindsay (19), respectively. The total protein content of tissues, which is necessary for the calculation of enzyme activities, was determined by the method of Lowry *et al*. (20).

***Analysis of mRNA transcript levels by real-time PCR (RT-PCR)***

The RT-PCR method was used to determine the mRNA transcript levels of significant genes involved in OS damage, inflammation damage, apoptotic damage, and autophagic damage. Total RNA isolation from rat lung tissues was performed using QIAzol Lysis Reagent (79306; Qiagen) and RNA content was measured with NanoDrop (BioTek Epoch) device. RNAs were then converted into cDNAs using iScript cDNA Synthesis Kit (Bio-Rad), and cDNA concentrations were measured with NanoDrop (BioTek Epoch). The relative mRNA transcript levels of the primer sequences shown in Table 1 were determined by Rotor-Gene Q (Qiagen) using iTaq Universal SYBR Green Supermix (BIO-RAD) according to the manufacturer’s instructions. The CT values obtained were normalized according to β-Actin using the 2^-deltadeltaCT^ method (21).

***Western blot analysis***

Western blot analysis of lung tissues was accomplished using the method of Ileriturk *et al*. (22). Lung tissues pulverized with liquid nitrogen were weighed and homogenized in RIPA buffer. Once homogenized, they were centrifuged (16,000g, 20 min). The total protein in the supernatants was determined using the Thermo FischerTM protein BCA assay kit. After protein measurement, supernatants were diluted with Laemmli sample buffer. The supernatants were separated by 10% sodium dodecyl sulfate-polyacrylamide gel electrophoresis with 30 µg protein in each well. Proteins separated by molecular size were transferred into PVDF membranes. After the proteins were transferred to the membranes, they were blocked with 4% Bovine serum albumin dissolved in phosphate-buffered saline with 0.1% tween (PBS-T) for 1.5 hr. Following this, proteins were incubated overnight together with the primary antibodies. At the end of incubation, goat anti-mouse IgG secondary antibody conjugated to HRP was incubated for 1.5 hr (1:2,000 dilution). After incubation with secondary antibodies, the membranes were washed five times with PBS-T for five min each time. The bands were then visualized with BioRad Clarity Max ECL substrate (Bio-Rad, Hercules, USA). Blots were subjected to densitometric analysis with the ImageLab program (Bio-Rad, Hercules, USA). At least three replicate measurements were taken.

***Histopathological evaluations***

Lung tissues were kept in a 10% neutral formalin buffer for 24 hr. After tissue follow-up, five μm thick sections were taken from the paraffin blocks with a microtome. The sections were placed on slides and stained with hematoxylin and eosin (H&E) stains. The stained samples were examined and photographed with an Olympus Cx 43 microscope (Japan).

***Statistical analysis***

Statistical analysis of the data obtained from lung tissues was performed with SPSS 20.0 (IBM, NY, USA) program. One-way ANOVA and Tukey’s *post hoc* tests were used for comparison between groups. In the RT-PCR method, each sample was run in triplicate. Data are presented as mean±SD. Statistical significance was accepted at three levels: *P*<0.05, *P*<0.01, and *P*<0.001.

## Results

***Effects of SA and ZNG administrations on oxidative stress***

**Figure 1:**
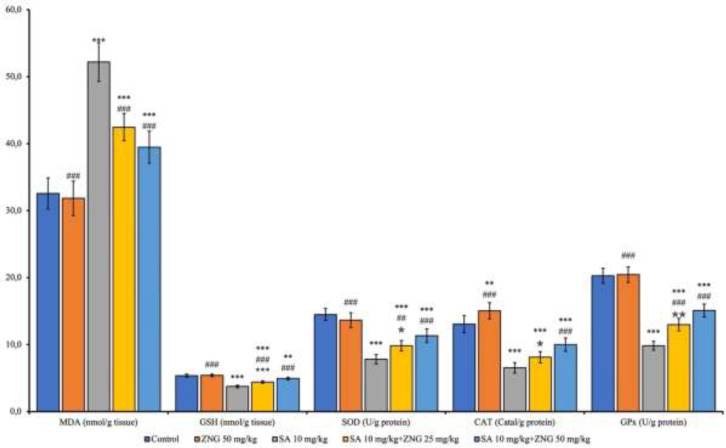
Lipid peroxidation and anti-oxidant levels in rat lung tissue

MDA levels, an indicator of LP, increased (*P*<0.001); and analyzed anti-oxidants decreased (*P*<0.001) with SA administration. Compared to the SA group, SOD (*P*<0.01), GPx, and GSH increased (*P*<0.001); MDA decreased (*P*<0.001) in the SA+ZNG 25mg/kg group. These effects were more in the SA+ZNG50mg/kg group (*P*<0.001 in all parameters) (Figure 1).

***Effects of SA and ZNG administrations on Nrf-2, HO-1, and NQO1 mRNA transcription***

**Figure 2:**
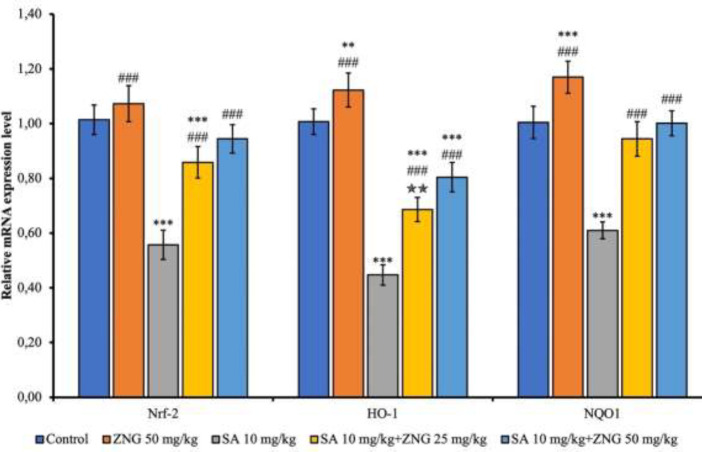
Nrf-2, HO-1, and NQO1 mRNA transcript levels in rat lung tissue Nrf-2: Nuclear factor erythroid 2–related factor 2, HO-1: Heme oxygenase 1, NQO1: NAD(P)H Quinone Dehydrogenase 1. Statistical significance was analyzed using One Way ANOVA (mean ± SD) (Control vs others: *P˂0.05, **P<0.01, ***P<0.001, SA10 mg/kg vs others: #*P*<0.05, ##*P*<0.01, ###*P*<0.001, SA 10 mg/kg+ZNG 25 mg/kg vs SA 10 mg/kg+ZNG 50 mg/kg: *P*˂0.05, *P*<0.01, *P*<0.001)

Compared to the control, Nuclear factor erythroid 2-related factor 2 (Nrf-2), Heme Oxygenase 1 (HO-1), and NAD(P)H Quinone Dehydrogenase 1 (NQO1) levels decreased in the SA group (*P*<0.001). Compared to the SA group, these parameters were increased in the SA+ZNG25mg/kg and SA+ZNG50mg/kg groups (*P*< 0.001). In 50 mg/kg dose compared to 25 mg/kg dose, this increase was even more significant in the HO-1 parameter (*P*<0.01) (Figure 2).

***Effects of SA and ZNG administrations on inflammation marker mRNA transcription***

**Figure 3:**
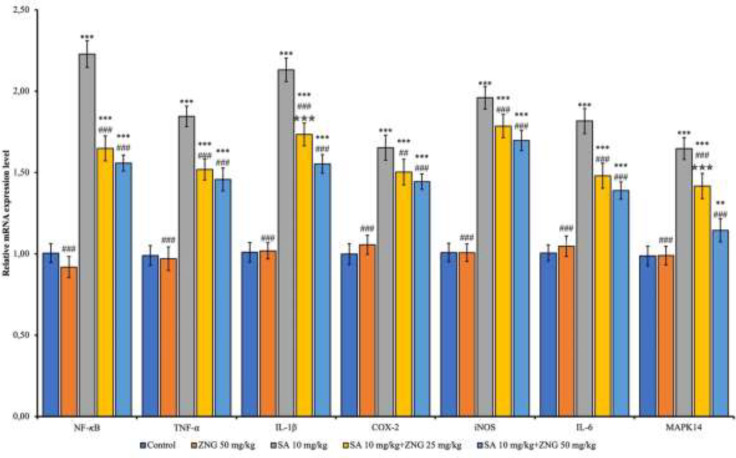
mRNA transcript levels of inflammation markers in the rat lung tissues

NF-κB, Tumor Necrosis Factor-α (TNF-α), Interleukin-1 beta (IL-1β), Cyclooxygenase-2 (COX-2), Inducible nitric oxide synthase (iNOS), Interleukin-6 (IL-6), and Mitogen-Activated Protein Kinase 14 (MAPK14) mRNA transcript levels were determined by RT-PCR method. Compared to the control, mRNA transcript levels of all these parameters increased in the SA group (*P*<0.001). COX-2 (*P*<0.01) and all other parameters (*P*<0.001) decreased in the SA+ZNG25mg/kg group compared to the SA group. Transcript levels of all these parameters decreased in SA+ZNG50mg/kg group compared to the SA group (*P*<0.001) (Figure 3).

***Effects of SA and ZNG administrations on apoptotic markers***

**Figure 4:**
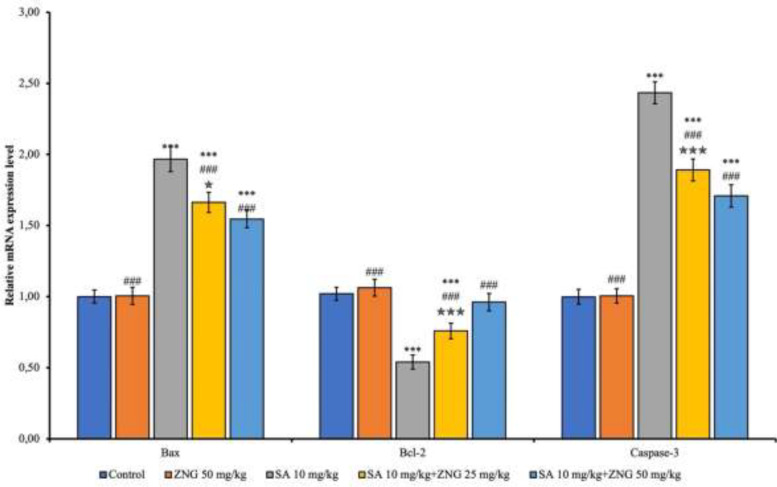
mRNA transcript level of apoptotic markers in the rat lung tissues

Apoptotic factors (Bax and Caspase-3) increased (*P*<0.001) and antiapoptotic factor B-cell lymphoma 2 (Bcl-2) decreased (*P*<0.001) with SA administration. SA+ZNG25mg/kg and SA+ZNG50mg/kg groups had decreased apoptotic factors and increased antiapoptotic factors compared to the SA group (*P*<0.001). The increase in Bcl-2 (*P*<0.001) and decrease in Bax (*P*<0.05) and Caspase-3 (*P*<0.001) were more pronounced at the higher dose (Figure 4).

***Effects of SA and ZNG administrations on autophagy markers***

**Figure 5:**
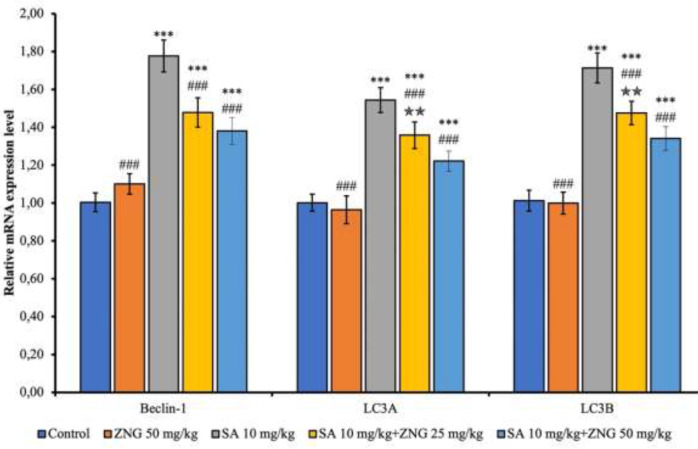
mRNA transcript levels of autophagy markers in the rat lung tissues

Beclin-1, Microtubule-associated protein 1 light chain 3 alpha (LC3A) and Microtubule-associated protein 1 light chain 3 beta (LC3B) mRNA transcript levels were measured to analyze the autophagy level in lung tissues. The mRNA transcription levels of Beclin-1, LC3A, and LC3B were increased by SA treatment (*P*<0.001). Transcript levels of these parameters decreased in SA+ZNG25mg/kg and SA+ZNG50 mg/kg groups compared to the SA group (*P*<0.001). When the doses were compared, the decrease in LC3A and LC3B parameters was higher at 50 mg/kg dose (*P*<0.01) (Figure 5).

***Effects of SA and ZNG administrations on IL-1β, NF-κB, and Beclin-1 protein levels***

**Figure 6:**
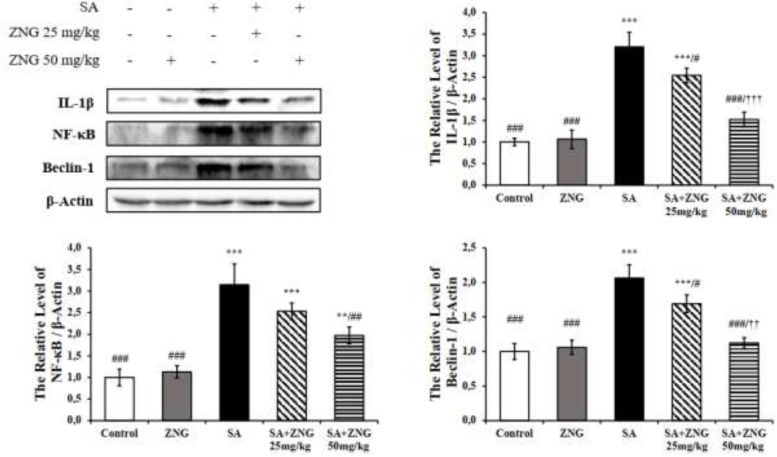
Effects of SA and ZNG administrations on IL-1β, NF-κB, and Beclin-1 protein levels in rat lung tissues

IL-1β, NF-κB, and Beclin-1 protein levels were increased with SA treatment (*P*<0.001) Compared to the SA group, it was found that IL-1β and Beclin-1 levels decreased in the SA+ZNG25mg/kg group (*P*<0.05), while IL-1β (*P*<0.001), NF-κB (*P*<0.01), and Beclin-1 (*P*<0.001) decreased in the SA+ZNG50mg/kg group (Figure 6).

***Histopathological findings***

**Figure 7:**
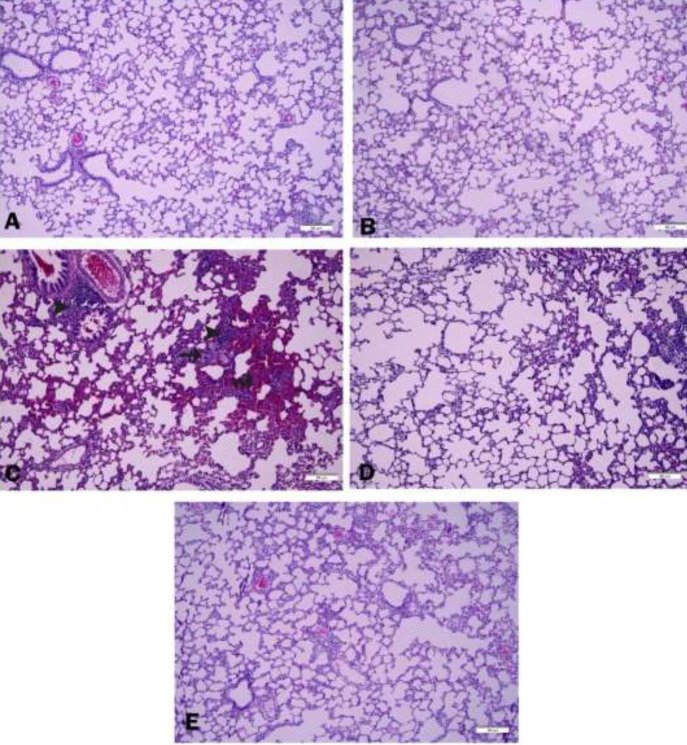
Photomicrographs of histological changes in lung tissue. (Hematoxylin-Eosin: H&E staining, scale bar: 50 μm)

Figure 7 shows the effects of ZNG on SA-induced histological changes in lung tissues using H&E staining. The lung tissues of both the control and ZNG groups had healthy histologic structures and no significant damage. In particular, alveolar septal thickness was normal, and bronchial and bronchial epithelial cells were arranged well (Figure 7A, 7B). When the sections in the SA group were examined, alveolar septal thickening and inflammatory cell infiltration were observed especially in the lung tissue. Hemorrhages and congestion in the interstitial space were particularly noticeable in this group. Some images showed alveolar enlargement, vacuoles, and edema. In conclusion, SA administration was found to impair lung morphology (Figure 7C). It was observed that the administration of ZNG in combination with SA reduced inflammatory cell infiltration and relieved hemorrhages in a dose-dependent manner. In this group, lung wall thickening was observed at a minimal level, especially at high doses (Figure 7D, 7E).

**Table 1:** The primer sequences of the related genes used in the RT-PCR (R: Reverse; F: Forward)

**Gene**	**Sequences (5’-3’)**	**Length (bp)**	**Accession no.**
**Nrf2**	F: TTTGTAGATGACCATGAGTCGC R: TCCTGCCAAACTTGCTCCAT	161	NM_031789.2
**HO-1**	F: ATGTCCCAGGATTTGTCCGA R: ATGGTACAAGGAGGCCATCA	144	NM_012580.2
**NQO1**	F: CTGGCCAATTCAGAGTGGCA R: GATCTGGTTGTCGGCTGGAA	304	NM_017000.3
**NF-** **B**	F: AGTCCCGCCCCTTCTAAAAC R: CAATGGCCTCTGTGTAGCCC	106	NM_001276711.1
**TNF-**	F: CTCGAGTGACAAGCCCGTAG R: ATCTGCTGGTACCACCAGTT	139	NM_012675.3
**IL-1**	F: ATGGCAACTGTCCCTGAACT R: AGTGACACTGCCTTCCTGAA	197	NM_031512.2
**COX-2**	F: AGGTTCTTCTGAGGAGAGAG R: CTCCACCGATGACCTGATAT	240	NM_017232.3
**iNOS**	F: AGATCAATGCAGCTGTGCTC R: GGCTCGATCTGGTAGTAGTAGA	235	NM_012611.3
**IL-6**	F: AGCGATGATGCACTGTCAGA R: GGAACTCCAGAAGACCAGAGC	127	NM_012589.2
**MAPK14**	F: GTGGCAGTGAAGAAGCTGTC R: GTCACCAGGTACACATCGTT	170	NM_031020.2
**Bax**	F: TTTCATCCAGGATCGAGCAG R: AATCATCCTCTGCAGCTCCA	154	NM_017059.2
**Bcl-2**	F: GACTTTGCAGAGATGTCCAG R: TCAGGTACTCAGTCATCCAC	214	NM_016993.2
**Caspase-3**	F: ACTGGAATGTCAGCTCGCAA R: GCAGTAGTCGCCTCTGAAGA	270	NM_012922.2
**Beclin-1**	F: TCTCGTCAAGGCGTCACTTC R: CCATTCTTTAGGCCCCGACG	198	NM_053739.2
**LC3A**	F: GACCATGTTAACATGAGCGA R: CCTGTTCATAGATGTCAGCG	139	NM_199500.2
**LC3B**	F: GAGCTTCGAACAAAGAGTGG R: CGCTCATATTCACGTGATCA	152	NM_022867.2
**-Actin**	F: CAGCCTTCCTTCTTGGGTATG R: AGCTCAGTAACAGTCCGCCT	360	NM_031144.3

MDA: Malondialdehyde, GSH: Glutathione, SOD: Superoxide dismutase, CAT: Catalase, GPx: Glutathione peroxidase

Statistical significance was analyzed using One Way ANOVA (mean ± SD) (Control vs others: *P˂0.05, **P<0.01, ***P<0.001, SA 10 mg/kg vs others: #*P*<0.05, ##*P*<0.01, ###*P*<0.001, SA 10 mg/kg+ZNG 25 mg/kg vs SA 10 mg/kg+ZNG 50 mg/kg: *P*˂0.05,*P*<0.01, *P*<0.001).

NF-κB: Nuclear factor kappa-B, TNF-α: tumor necrosis factor-alpha, IL-1β: interleukin-1 beta, COX-2: cyclooxygenase-2, iNOS: inducible nitric oxide synthase, IL-6: Interleukin-6. Statistical significance was analyzed using One Way ANOVA (mean ± SD) (Control vs others: *P˂0.05, **P<0.01, ***P<0.001, SA 10 mg/kg vs others: #*P*<0.05, ##*P*<0.01, ###*P*<0.001, SA 10 mg/kg+ZN G25 mg/kg vs SA 10 mg/kg+ZNG 50 mg/kg: *P*˂0.05,*P*<0.01,*P*<0.001)

Bcl-2: B-cell lymphoma 2, Caspase-3: cysteine–aspartic acid protease. Statistical significance was analyzed using One Way ANOVA (mean ± SD) (Control vs others: *P˂0.05, **P<0.01, ***P<0.001, SA10mg/kg vs others: #*P*<0.05, ##*P*<0.01, ###*P*<0.001, SA10 mg/kg+ZNG25 mg/kg vs SA10 mg/kg+ZNG50 mg/kg: *P*˂0.05, *P*<0.01, *P*<0.001).

LC3A: Microtubule-associated protein 1 light chain 3 alpha, LC3B: Microtubule-associated protein 1 light chain 3 beta. Statistical significance was analyzed using One Way ANOVA (mean ± SD) (Control vs others: *P˂0.05, **P<0.01, ***P<0.001, SA 10 mg/kg vs others: #*P*<0.05, ##*P*<0.01, ###*P*<0.001, SA 10 mg/kg+ZNG25 mg/kg vs SA 10 mg/kg+ZNG 50 mg/kg: *P*˂0.05, *P*<0.01, *P*<0.001).

NF-κB: Nuclear factor kappa-B, IL-1β: interleukin-1 beta. Statistical significance was analyzed using One Way ANOVA (mean ± SD) (Control vs others: *P˂0.05, **P<0.01, ***P<0.001, SA 10 mg/kg vs others: #*P*<0.05, ##*P*<0.01, ###*P*<0.001, SA 10 mg/kg+ZNG 25 mg/kg vs SA10 mg/kg+ZNG 50 mg/kg: *P*˂0.05, P<0.01,P<0.001).

A: Control, B: ZNG50 mg/kg; Zingerone group, C: SA10 mg/kg; Sodium arsenite group; arrowhead: infiltration of inflammatory cells, curved arrow: bleeding, arrow: thickening of alveolar wall, D: SA+ZNG 25 mg/kg group; E: SA+ZNG 50 mg/kg group

## Discussion

Among environmental pollutants, AR is found all over the world and still affects tens of millions of people worldwide. There are two most dangerous inorganic forms of AR; arsenite and arsenate (23). The maximum acceptable level of AR in drinking water is 0.01 mg/l. However, higher levels have been reported in many drinking waters worldwide, and the more hazardous inorganic forms are more prevalent in drinking water (24). Long and short-term exposure to inorganic forms affects lung tissue (23). An important aspect pointed out in the literature to combat AR-induced body toxicity is the administration of anti-oxidants (25). ZNG is an alkanone with potent anti-oxidant properties that is the active component of ginger (26). As a result, ZNG was chosen as an anti-oxidant in the current study, and the effects of ZNG on OS, inflammation, apoptotic, and autophagy damages in SA-induced rat lung tissue toxicity were investigated by biochemical, molecular, and histopathological methods.

OS occurs when ROS increase and anti-oxidants decrease (27). Lipids, an essential component of the cell membrane, are affected by the increase in ROS. Therefore, ROS increase causes LP. MDA, the end product of multiple fatty acid metabolism (28), is a significant marker of LP and increased OS. Anti-oxidant enzymes CAT, SOD, and GPx reduce OS damage (29). GSH is another protective mechanism against OS by binding ROS products (30 Increased MDA levels in lung tissues of people who drink AR-contaminated water for long periods, together with decreased GSH levels and CAT activity, lead to poor OS and, ultimately, OS-induced DNA strand breakage (31). The most prominent feature of OS in AR toxicity is the increase in LP and the main indicator of this is the increase in MDA (25). When the current study’s OS findings were considered together, SA increased OS by increasing MDA levels and decreasing anti-oxidant enzyme activities in lung tissues. ZNG administration, on the other hand, increased anti-oxidant activity by decreasing SA-induced increased MDA levels and increasing SOD, CAT, and GPx activities. This anti-oxidant activity was stronger, especially at 50 mg/kg dose. Mohemmed reported that ZNG decreased MDA levels by reducing LP and had a protective effect on tissue, which is similar to our findings (13). Kandemir *et al*. reported that ZNG increased anti-oxidant capacity by increasing GSH levels and SOD, CAT, and GPx activities and decreased MDA levels, which is consistent with the data of our study (10). The reason for this effect of ZNG may be a result of its ability to transfer electrons, chelate metals, and scavenge arsenite-induced free radicals by reactivating decreased anti-oxidant enzymes.

In addition to deactivating the anti-oxidant capacity in the body, increased ROS can also impair post-translational protein activities. Moreover, it can affect gene activities by regulating important transcription factors and promoting an inflammatory environment in addition to OS (32). This effect of excessive ROS production is a significant factor in its acting as a signaling molecule to activate NF-κB. Once NF-kB is activated, it translocates toward the nucleus to alter the expression of inflammation-related COX-2 and TNF-α genes (33). Studies have reported that COX-2 plays a role in many physiological activities. Different mediators associated with inflammation such as growth factors, lipopolysaccharide, interleukin-1β, and TNF-α affect COX-2 expression (34). TNF-α is a significant immune response factor that initiates and regulates the cytokine cascade (35). COX-2, TNF-α, and iNOS all play significant roles in inflammation and their activation is mediated by NF-κB (36). Therefore, inhibiting NF-κB is therapeutically important in preventing inflammation (37). In the current study, SA caused inflammatory damage in lung tissues by increasing NF-κB and related proinflammatory cytokines. ZNG administration, on the other hand, reduced SA-induced inflammatory damage by reducing NF-κB and related proinflammatory cytokines. ZNG could emerge as an effective therapeutic agent in lung toxicity caused by inflammation due to SA exposure. Similar to our study, it was reported that SA administration triggered NF-κB activation and increased proinflammatory substances such as COX-2 and iNOS, resulting in inflammatory damage (38). On the other hand, consistent with the findings in our study, Akaras *et al*. reported that ZNG administration decreased tissue inflammation by inhibiting NF-κB activity in SA-induced nephrotoxicity (14). The reason for ZNG’s anti-inflammatory effect could be that it tries to reduce the development of inflammation by decreasing ROS production and increasing anti-oxidant gene activation. Because reducing ROS generation prevents NF-κB translocation to the nucleus and subsequently proinflammatory cytokines will not be stimulated.

MAPK14, also known as p38α, is known as another key regulator of the inflammatory response (39). The MAPK signaling pathway also activates the inflammation-associated NF-κB signaling pathway (14, 40). In this study, SA significantly increased MAPK14 activity. In their study with different toxic agents, A study showed that MAPK14 increased lung toxicity, similar to this study (41). The activity of this enzyme was significantly reduced following ZNG administration. It can be stated that SA significantly triggers inflammation by activating both pathways. ZNG showed an anti-inflammatory effect in this SA-induced lung toxicity with its inhibitory effect on both pathways.

Apoptosis occurs due to DNA or protein damage resulting from excessive ROS production (42, 43). Apoptosis is a process that eliminates cells in the body that are harmful or need to be destroyed. However, if it increases, it causes stress or damage to healthy cells as well (44, 45). Bax is an apoptotic factor and opens membrane pores in mitochondria. Bcl-2 is an antiapoptotic factor that prevents the opening of these pores. Once caspase-3 is active, the irreversible step in the apoptotic process is initiated, which causes proteolytic degradation and death of cells (46). In this study, mRNA transcript levels of Caspase-3 and Bax, which are apoptotic factors, increased with SA exposure in lung tissues, while Bcl-2, which is an antiapoptotic factor, decreased. When ZNG was administered together with SA, the opposite effect was observed and ZNG exhibited antiapoptotic properties. Therefore, ZNG may be an effective agent against apoptosis in SA-induced lung toxicity.

Autophagy is another pathway caused by SA-induced OS (47). Autophagy is a protective mechanism that removes dysfunctional or senescent organelles via lysosomes. In physiological conditions, autophagy plays an active role in cellular homeostasis and occurs at low levels. However, when overstimulated, it brings with it the loss of healthy cells and tissues (48). In this study, Beclin-1 mRNA transcription and protein level, a significant autophagy marker, increased with SA application. This indicates that SA triggers autophagy. ZNG, on the other hand, when combined with SA, showed an antiautophagic effect and decreased Beclin-1 levels. Similar to our study, it was reported that ZNG showed anti autophagic effect by decreasing the Beclin-1 level (49).

Nrf2 is a critical transcription factor co-stimulated by OS. Nrf2 is effective in the regulation of some anti-oxidant genes (50). Once activated, Nrf-2 is transported to the nucleus and regulates the activation of HO-1 and NQO1 (51). HO-1 is one of the target genes of Nrf-2. Increased HO-1 enzyme activity is a potent protective mechanism against ROS-induced OS damage (52, 53). Nrf-2 also initiates the activation of the NQO1 protein. NQO1 protein functions include protecting against natural and exogenous quinones, activating endogenous anti-oxidants, and stabilizing the p53 protein (54). Once the Nrf-2 signaling pathway is active, it can effectively contribute to tissue healing, primarily by preventing OS damage (55). Nrf-2, on the other hand, can directly stop cellular apoptosis by stimulating the expression of BCL-xL (54). For these reasons, Nrf-2 is essential for the relief of lung disorders, and lung tissue pathological processes can be effectively alleviated by activation of Nrf-2 (56). Nrf-2-deficient rats tend to suffer from diseases caused by oxidants and toxic substances, for example, pulmonary damage induced by inhaled AR (51). In this study, Nrf-2, HO-1, and NQO1 levels were decreased by SA exposure in rat lung tissues. Similar to our study, it was reported that Nrf-2, HO-1, and NQO1 parameters decreased with the toxic agent in a different tissue in the body (57). The opposite was observed to increase when ZNG was administered together with SA. This suggests that ZNG causes an increase in anti-oxidant capacity through the Nrf-2 pathway.

Exposure of the human body to SA is usually by ingestion or inhalation. Thus, lung tissue is one of the target tissues of SA exposure and a sensitive one (32). Even acute exposure to different doses of AR causes decreased cell viability and morphological changes (58). The epithelial cell is the main cell type in the lung and is one of the critical structures affected by lung diseases. In a study, arsenite administration increased collagen fiber deposition in the peri-bronchioles and alveolar region. They reported that AR exposure may trigger both obstructive and restrictive lung diseases (59). Hemorrhages and congestion were remarkable in the tissues administered with SA in our study, especially in the interstitial area. However, it was found that these hemorrhages decreased dose-dependently when ZNG was administered. We found that lung thickening, which increased with SA administration, regressed with ZNG administration. It was remarkable that this thickening was reduced to a minimal level at the dose of 50 mg/kg. In general, it can be stated that SA administration disrupted lung morphology and ZNG administration was used to preserve the morphology.

## Conclusion

In summary, ZNG reduced SA-induced toxic damage by reducing OS damage, inflammation damage, apoptotic and autophagic cell death, and activating anti-oxidant defense systems in rat lung tissues. The present study supports the hypothesis that ZNG may have a protective effect in SA-induced damage to rat lung tissues.

## Authors’ Contributions

All authors contributed to the concept and design of this study, also to material preparation, data collection, and analysis. All authors have read and approved the article written by H Ş.

## Etical Approval

Ethical approval was obtained by Atatürk University Animal Experiments Local Ethics Committee (No: 2022/11-238, Date: 27.10.2022).

## Consent to Participate

All authors have given their explicit consent for the publication of the manuscript. Hasan Şimşek has the consent of all authors for contact by anyone for further clarification and information about the research if necessary.

## Conflicts of Interest

The authors have no conflicts of interest.
